# 6-Amino-1,3-dimethyl-5-[(*E*)-2-(methyl­sulfan­yl)benzyl­idene­amino]­pyrimidine-2,4(1*H*,3*H*)-dione–1,3,7,9-tetra­methyl­pyrimido[5,4-*g*]pteridine-2,4,6,8-tetrone (1/1)

**DOI:** 10.1107/S1600536812029716

**Published:** 2012-07-04

**Authors:** Irvin N. Booysen, Muhammed B. Ismail, Matthew P. Akerman

**Affiliations:** a University of KwaZulu-Natal, School of Chemistry and Physics, Private Bag X01, Scottsville 3209, Pietermaritzburg, South Africa

## Abstract

In the title co-crystal, C_12_H_12_N_6_O_4_·C_14_H_16_N_4_O_2_S, both mol­ecules are essentially planar [maximum deviations = 0.129 (1) and 0.097 (1) Å, respectively]. The tricyclic and Schiff base mol­ecules are alternately stacked along the *a* axis and are linked by π–π inter­actions with centroid–centroid distances of 3.5170 (16) and 3.6576 (17) Å. An intra­molecular C—H⋯O hydrogen bond and a C—H⋯S contact occur in the Schiff base molecule. In the crystal, N—H⋯O, N—H⋯N and C—H⋯O hydrogen bonds lead to the formation of a three-dimensional network.

## Related literature

For the crystal structure of a Schiff base derived from 5,6-diamino-1,3-dimethyl­pyrimidine-2,4(1*H*,3*H*)-dione and pico­lin­aldehyde, see: Booysen *et al.* (2011*a* ▶). For the crystal structure of the title Schiff base, see: Booysen *et al.* (2011*b* ▶). For the crystal structure of the title tricyclic compound, see: Booysen *et al.* (2008 ▶). For details of the use of the Schiff base *N*-(2-amino­benzyl­idene)-5-amino-1,3-dimethyl­pyrimidine-2,4(1*H*,3*H*)-dione as a che­lating ligand towards rhenium, see: Mayer *et al.* (2010 ▶). For applications of Schiff base ligands, see: Kumar *et al.*. 2009 ▶.
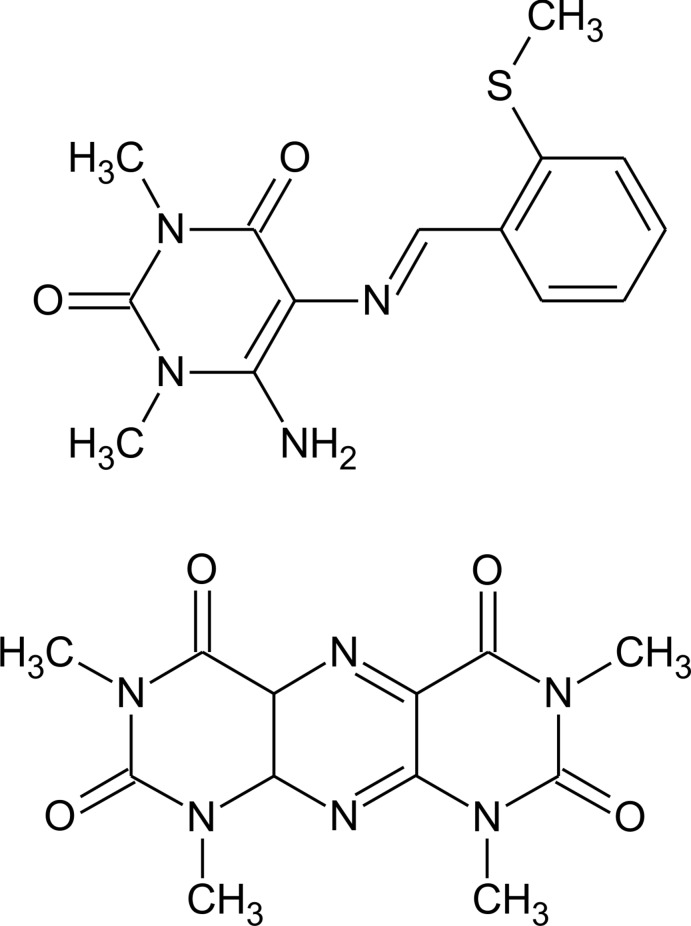


## Experimental

### 

#### Crystal data

C_12_H_12_N_6_O_4_·C_14_H_16_N_4_O_2_S
*M* *_r_* = 608.64
Monoclinic, 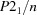

*a* = 6.8501 (9) Å
*b* = 25.594 (4) Å
*c* = 15.284 (2) Å
β = 99.315 (5)°
*V* = 2644.3 (7) Å^3^
*Z* = 4
Mo *K*α radiation
μ = 0.19 mm^−1^
*T* = 100 K
0.30 × 0.05 × 0.05 mm

#### Data collection

Bruker APEXII CCD diffractometer
Absorption correction: multi-scan (*SADABS*; Bruker 2010 ▶) *T*_min_ = 0.946, *T*_max_ = 0.991
14953 measured reflections
5846 independent reflections
4076 reflections with *I* > 2σ(*I*)
*R*_int_ = 0.057

#### Refinement

*R*[*F* ^2^ > 2σ(*F* ^2^)] = 0.060
*wR*(*F* ^2^) = 0.171
*S* = 1.01
5846 reflections
403 parameters
H atoms treated by a mixture of independent and constrained refinement
Δρ_max_ = 0.42 e Å^−3^
Δρ_min_ = −0.39 e Å^−3^

### 

Data collection: *APEX2* (Bruker, 2010 ▶); cell refinement: *SAINT-Plus* (Bruker, 2010 ▶); data reduction: *SAINT-Plus*; program(s) used to solve structure: *SHELXS97* (Sheldrick, 2008 ▶); program(s) used to refine structure: *SHELXL97* (Sheldrick, 2008 ▶); molecular graphics: *WinGX* (Farrugia, 1999 ▶); software used to prepare material for publication: *publCIF* (Westrip, 2010 ▶).

## Supplementary Material

Crystal structure: contains datablock(s) I, global. DOI: 10.1107/S1600536812029716/rz2778sup1.cif

Structure factors: contains datablock(s) I. DOI: 10.1107/S1600536812029716/rz2778Isup2.hkl

Supplementary material file. DOI: 10.1107/S1600536812029716/rz2778Isup3.cml

Additional supplementary materials:  crystallographic information; 3D view; checkCIF report

## Figures and Tables

**Table 1:** Hydrogen-bond geometry (Å, °)

*D*—H⋯*A*	*D*—H	H⋯*A*	*D*⋯*A*	*D*—H⋯*A*
N2*A*—H101⋯N1*A*	0.85 (4)	2.32 (3)	2.701 (4)	107 (2)
N2*A*—H101⋯O2*B* ^i^	0.85 (4)	2.37 (3)	3.078 (3)	141 (3)
N2*A*—H102⋯O4*A* ^i^	0.96 (4)	2.29 (4)	3.238 (3)	169 (3)
C4*A*—H2⋯O2*A* ^ii^	0.95	2.58	3.159 (4)	119
C3*A*—H3⋯O2*A* ^ii^	0.95	2.49	3.114 (4)	123
C12*A*—H2′3⋯O4*A* ^i^	0.98	2.36	3.014 (4)	123
C1*A*—H5⋯O2*B* ^i^	0.95	2.45	3.339 (4)	155
C7*A*—H7⋯S1	0.95	2.59	3.017 (3)	108
C7*A*—H7⋯O1*A*	0.95	2.18	2.849 (3)	127
C13*B*—H5*A*1⋯O2*B* ^i^	0.98	2.50	3.315 (4)	140
C10*B*—H6*A*1⋯O1*A* ^iii^	0.98	2.58	3.542 (4)	169

Symmetry codes: (i) 
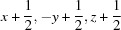
; (ii) 
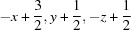
; (iii) 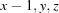.

**Fig. 1.**
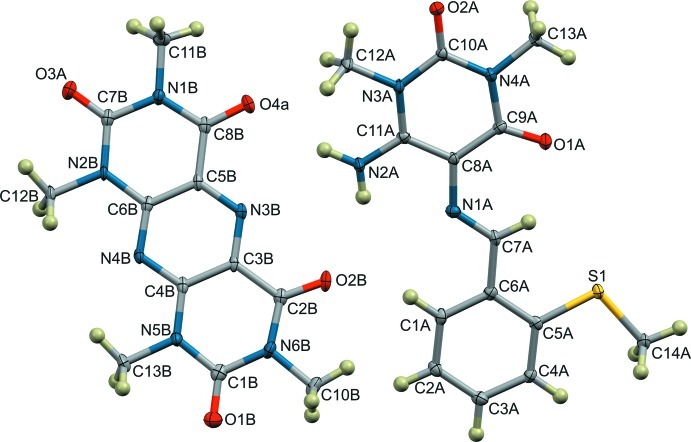
The asymmetric unit of the title compound. Displacement ellipsoids have been rendered at the 50% probability level.

**Fig. 2.**
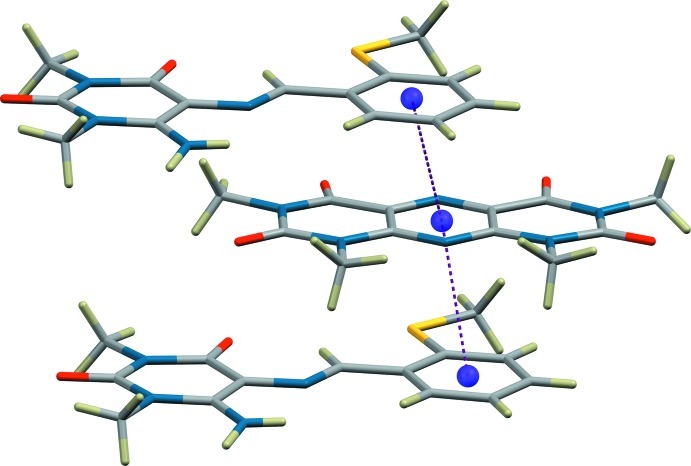
The tricyclic molecule sandwiched between two Schiff base molecules. π -π interactions are shown as dashed lines.

**Fig. 3.**
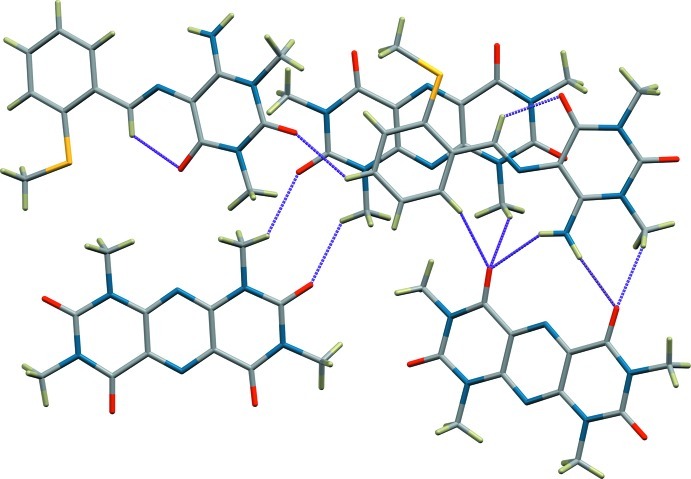
Hydrogen bonding between the tricyclic molecule and the Schiff base molecule.

## supplementary crystallographic information

### Comment

Schiff bases and their metal complexes exhibit unique properties which make them favourable for an array of applications such as catalysis and medicinal chemistry (Kumar *et al.*, 2009). Moreover, Schiff bases have the added advantage of being readily derivatized and their imino nitrogen atoms possess high thermodynamic stability towards various metal centres. In an attempt, to afford a novel rhenium(I) complex containing the Schiff base, C_14_H_16_N_4_O_2_S, the title compound was unexpectedly isolated. The asymmetric unit consists of the Schiff base molecule and a tricyclic molecule, (Fig. 1) which are planar. The tricyclic molecule has been previously isolated by the oxidative deamination of 5,6-diamino-1,3-dimethylpyrimidine-2,4(1*H*, 3*H*)-dione by ammonium perrhenate (Booysen *et al.*, 2008).

In the Schiff base molecule, the *ortho*-(thiomethyl) phenyl moiety is nearly co-planar with the 6-amino-1,3-dimethylpyrimidine-2,4(1*H*, 3*H*)-dione moiety. The largest deviation from the 21-atom mean plane defined by all non-hydrogen atoms of the Schiff base molecule is 0.097 (1) Å exhibited by the primary amine nitrogen atom. The C7—N1A bond length, 1.282 (4) Å, and the C8A—N1A—C7A bond angle, 124.5 (3)°, emphasize the *sp*^2^ hybridization of the imino nitrogen atom. An *E*-configuration is observed for the Schiff base moiety.

The tricyclic molecule consists of three fused rings, a central pyrazine ring and two terminal pyrimidine rings. The largest deviation from planarity is shown by the methyl carbon atom C10B, which is displaced by 0.129 (1) Å from the 22-atom mean plane defined by all non-hydrogen atoms. The bond lengths within the pyrazine ring suggest that they are part of a *π-*delocalized ring system. Thus the N4B and N5B atoms are *sp*^2^-hybridized which is confirmed by the bond angles C5B—N5B—C3B [117.2 (2)°] and C6B—N4B—C4B [115.6 (2)°].

The two molecules exhibit *π-π* interactions. Each tricyclic molecule is sandwiched between two Schiff base molecules. The distance between the centroid of the pyrazine ring, *Cg*1, and the centroid of the Schiff base phenyl ring, *Cg*2, within the aysmmetric unit is 3.517 (1) Å. The distance between *Cg*1 and *Cg*2 of a symmetry related molecule is 3.658 (1) Å (Figure 2). The crystal lattice is further stablized by a large number of both classical and non-classical hydrogen bonds. These hydrogen bonds are both inter and inramolecular in nature and ultimately link the Schiff base and tricyclic molecules into an infinite, three-dimensional network. Hydrogen bond lengths and angles are summarized in Table 1. The hydrogen bonding pattern is illustrated in Figure 3.

### Experimental

The title compound was prepared from the reaction of [Re(CO_5_Br] (100 mg, 245 µmol) and 6-amino-1,3-dimethyl-5-[(*E*)-2-(methylsulfanyl)benzylideneamino]pyrimidine-2,4(1*H*,3*H*)-dione (149 mg, 490 µmol) in refluxing toluene (20 cm^3^) for three hours under nitrogen. X-ray quality yellow crystals were grown from the slow evaporation of the mother liquor.

### Refinement

All non-hydrogen atoms were located in the difference Fourier map and refined anisotropically. The positions of all hydrogen atoms were calculated using the standard riding model of *SHELXL97* with C—H(aromatic) distances of 0.93 Å and *U*_iso_ = 1.2 *U*_eq_, and *C*—*H*(methyl) distances of 0.96 Å and *U*_iso_ = 1.5 *U*_eq_. The amine hydrogen atoms were located in the difference Fourier map and allowed to refine isotropically.

### Crystal data

**Table:**

C_12_H_12_N_6_O_4_·C_14_H_16_N_4_O_2_S	*F*(000) = 1272
*M**_r_* = 608.64	*D*_x_ = 1.529 Mg m^−^^3^
Monoclinic, *P*2_1_/*n*	Mo *K*α radiation, λ = 0.71073 Å
Hall symbol: -P 2yn	Cell parameters from 4076 reflections
*a* = 6.8501 (9) Å	θ = 1.6–27.3°
*b* = 25.594 (4) Å	µ = 0.19 mm^−^^1^
*c* = 15.284 (2) Å	*T* = 100 K
β = 99.315 (5)°	Needle, yellow
*V* = 2644.3 (7) Å^3^	0.30 × 0.05 × 0.05 mm
*Z* = 4	

### Data collection

**Table:**

Bruker APEXII CCD diffractometer	5846 independent reflections
Radiation source: fine-focus sealed tube	4076 reflections with *I* > 2σ(*I*)
Graphite monochromator	*R*_int_ = 0.057
ω and φ scans	θ_max_ = 27.3°, θ_min_ = 1.6°
Absorption correction: multi-scan (*SADABS*; Bruker 2010)	*h* = −4→8
*T*_min_ = 0.946, *T*_max_ = 0.991	*k* = −32→25
14953 measured reflections	*l* = −19→18

### Refinement

**Table:**

Refinement on *F*^2^	Primary atom site location: structure-invariant direct methods
Least-squares matrix: full	Secondary atom site location: difference Fourier map
*R*[*F*^2^ > 2σ(*F*^2^)] = 0.060	Hydrogen site location: inferred from neighbouring sites
*wR*(*F*^2^) = 0.171	H atoms treated by a mixture of independent and constrained refinement
*S* = 1.01	*w* = 1/[σ^2^(*F*_o_^2^) + (0.0805*P*)^2^ + 2.8029*P*] where *P* = (*F*_o_^2^ + 2*F*_c_^2^)/3
5846 reflections	(Δ/σ)_max_ = 0.021
403 parameters	Δρ_max_ = 0.42 e Å^−^^3^
0 restraints	Δρ_min_ = −0.39 e Å^−^^3^

### Special details

**Table:**

Geometry. All s.u.'s (except the s.u. in the dihedral angle between two l.s. planes) are estimated using the full covariance matrix. The cell s.u.'s are taken into account individually in the estimation of s.u.'s in distances, angles and torsion angles; correlations between s.u.'s in cell parameters are only used when they are defined by crystal symmetry. An approximate (isotropic) treatment of cell s.u.'s is used for estimating s.u.'s involving l.s. planes.
Refinement. Refinement of *F*^2^ against ALL reflections. The weighted *R*-factor *wR* and goodness of fit *S* are based on *F*^2^, conventional *R*-factors *R* are based on *F*, with *F* set to zero for negative *F*^2^. The threshold expression of *F*^2^ > 2σ(*F*^2^) is used only for calculating *R*-factors(gt) *etc*. and is not relevant to the choice of reflections for refinement. *R*-factors based on *F*^2^ are statistically about twice as large as those based on *F*, and *R*- factors based on ALL data will be even larger.

### Fractional atomic coordinates and isotropic or equivalent isotropic displacement parameters (Å^2^)

**Table:**

	*x*	*y*	*z*	*U*_iso_*/*U*_eq_	
S1	0.72820 (11)	0.31430 (3)	0.09858 (5)	0.01445 (19)	
N1A	0.7048 (4)	0.19781 (9)	0.29893 (16)	0.0137 (5)	
C6B	0.3512 (4)	0.38704 (11)	0.29375 (18)	0.0122 (6)	
C10A	0.5483 (4)	0.04004 (11)	0.24893 (18)	0.0117 (6)	
O1B	0.1720 (3)	0.16717 (9)	0.27878 (15)	0.0216 (5)	
C9A	0.6023 (4)	0.12732 (11)	0.18728 (19)	0.0123 (6)	
O2B	0.1935 (3)	0.26945 (8)	0.03637 (13)	0.0185 (5)	
C2B	0.2047 (4)	0.26570 (12)	0.11659 (19)	0.0138 (6)	
N3A	0.5985 (4)	0.06106 (9)	0.33332 (15)	0.0130 (5)	
O3A	0.4773 (3)	0.51453 (8)	0.37187 (14)	0.0226 (5)	
N2B	0.3985 (4)	0.42849 (10)	0.35067 (15)	0.0141 (5)	
O4A	0.3461 (3)	0.45658 (8)	0.08667 (13)	0.0204 (5)	
N1B	0.4176 (4)	0.48549 (10)	0.22915 (16)	0.0140 (5)	
C12A	0.5942 (5)	0.02564 (12)	0.4084 (2)	0.0200 (7)	
H2'1	0.5327	−0.0075	0.3869	0.030*	
H2'2	0.5174	0.0416	0.4502	0.030*	
H2'3	0.7296	0.0191	0.4384	0.030*	
N4A	0.5536 (3)	0.07345 (9)	0.17905 (15)	0.0130 (5)	
N6B	0.1685 (4)	0.21905 (10)	0.15727 (16)	0.0154 (5)	
N3B	0.2817 (3)	0.35639 (10)	0.14445 (15)	0.0130 (5)	
O2A	0.5003 (3)	−0.00589 (8)	0.23777 (13)	0.0160 (5)	
C12B	0.4159 (5)	0.42088 (13)	0.44709 (19)	0.0194 (7)	
H8A1	0.3139	0.3964	0.4595	0.029*	
H8A2	0.3985	0.4545	0.4756	0.029*	
H8A3	0.5470	0.4067	0.4704	0.029*	
C13B	0.2677 (5)	0.24331 (13)	0.39999 (19)	0.0219 (7)	
H5A1	0.3921	0.2248	0.4201	0.033*	
H5A2	0.1566	0.2220	0.4125	0.033*	
H5A3	0.2684	0.2767	0.4313	0.033*	
C13A	0.5062 (5)	0.05017 (12)	0.09037 (18)	0.0176 (7)	
H1'1	0.5963	0.0210	0.0854	0.026*	
H1'2	0.5212	0.0766	0.0455	0.026*	
H1'3	0.3695	0.0374	0.0810	0.026*	
O1A	0.5986 (3)	0.15304 (8)	0.11890 (13)	0.0185 (5)	
N4B	0.3251 (4)	0.34063 (9)	0.32905 (15)	0.0125 (5)	
C5B	0.3307 (4)	0.39547 (11)	0.20163 (18)	0.0124 (6)	
N2A	0.7067 (4)	0.12816 (11)	0.43183 (17)	0.0173 (6)	
N5B	0.2466 (4)	0.25296 (10)	0.30381 (15)	0.0146 (5)	
C11A	0.6542 (4)	0.11251 (11)	0.34771 (18)	0.0118 (6)	
C3A	0.8699 (4)	0.38866 (12)	0.33665 (19)	0.0162 (6)	
H3	0.9055	0.4228	0.3579	0.019*	
C7A	0.7108 (4)	0.23495 (11)	0.24313 (18)	0.0128 (6)	
H7	0.6804	0.2280	0.1814	0.015*	
C5A	0.7767 (4)	0.32939 (11)	0.21317 (18)	0.0118 (6)	
C8B	0.3641 (4)	0.44696 (12)	0.16650 (19)	0.0146 (6)	
C7B	0.4332 (4)	0.47830 (12)	0.32141 (19)	0.0151 (6)	
C1B	0.1943 (4)	0.20993 (12)	0.2492 (2)	0.0164 (6)	
C8A	0.6539 (4)	0.14645 (11)	0.27595 (18)	0.0116 (6)	
C2A	0.8602 (4)	0.34814 (12)	0.39644 (19)	0.0153 (6)	
H4	0.8905	0.3544	0.4584	0.018*	
C6A	0.7642 (4)	0.28798 (11)	0.27399 (18)	0.0126 (6)	
C4A	0.8276 (4)	0.37937 (11)	0.24528 (19)	0.0131 (6)	
H2	0.8336	0.4073	0.2049	0.016*	
C1A	0.8059 (4)	0.29850 (12)	0.36480 (19)	0.0143 (6)	
H5	0.7968	0.2711	0.4058	0.017*	
C3B	0.2553 (4)	0.30973 (11)	0.17811 (18)	0.0116 (6)	
C4B	0.2761 (4)	0.30162 (11)	0.27104 (18)	0.0125 (6)	
C11B	0.4385 (5)	0.53951 (12)	0.1995 (2)	0.0190 (7)	
H9A1	0.4701	0.5394	0.1392	0.028*	
H9A2	0.5452	0.5568	0.2396	0.028*	
H9A3	0.3142	0.5584	0.2000	0.028*	
C10B	0.1018 (5)	0.17369 (12)	0.1007 (2)	0.0211 (7)	
H6A1	−0.0311	0.1634	0.1100	0.032*	
H6A2	0.1934	0.1445	0.1163	0.032*	
H6A3	0.0989	0.1830	0.0384	0.032*	
C14A	0.7605 (5)	0.37598 (12)	0.0459 (2)	0.0202 (7)	
H20A	0.6672	0.4016	0.0631	0.030*	
H20B	0.7358	0.3715	−0.0186	0.030*	
H20C	0.8961	0.3884	0.0647	0.030*	
H101	0.732 (5)	0.1605 (14)	0.439 (2)	0.020 (9)*	
H102	0.737 (6)	0.1055 (16)	0.482 (3)	0.040 (11)*	

### Atomic displacement parameters (Å^2^)

**Table:**

	*U*^11^	*U*^22^	*U*^33^	*U*^12^	*U*^13^	*U*^23^
S1	0.0197 (4)	0.0154 (4)	0.0089 (4)	−0.0009 (3)	0.0042 (3)	−0.0001 (3)
N1A	0.0140 (13)	0.0124 (13)	0.0152 (13)	−0.0009 (10)	0.0035 (9)	−0.0011 (10)
C6B	0.0082 (13)	0.0170 (15)	0.0112 (14)	0.0029 (11)	0.0010 (10)	−0.0040 (11)
C10A	0.0116 (14)	0.0135 (15)	0.0106 (14)	0.0021 (11)	0.0035 (10)	0.0014 (11)
O1B	0.0240 (12)	0.0175 (12)	0.0230 (12)	0.0009 (9)	0.0031 (9)	0.0015 (9)
C9A	0.0106 (14)	0.0120 (15)	0.0148 (15)	0.0000 (11)	0.0039 (10)	0.0015 (11)
O2B	0.0192 (11)	0.0264 (12)	0.0095 (10)	−0.0005 (9)	0.0011 (8)	−0.0064 (9)
C2B	0.0092 (14)	0.0191 (16)	0.0130 (15)	0.0007 (11)	0.0015 (10)	−0.0030 (11)
N3A	0.0164 (13)	0.0147 (13)	0.0080 (12)	−0.0017 (10)	0.0022 (9)	0.0020 (9)
O3A	0.0314 (13)	0.0205 (12)	0.0154 (12)	−0.0035 (10)	0.0022 (9)	−0.0066 (9)
N2B	0.0186 (13)	0.0175 (13)	0.0054 (12)	0.0016 (10)	−0.0005 (9)	−0.0038 (9)
O4A	0.0287 (13)	0.0236 (12)	0.0087 (11)	−0.0002 (10)	0.0024 (9)	0.0020 (9)
N1B	0.0134 (13)	0.0171 (13)	0.0116 (13)	−0.0026 (10)	0.0025 (9)	−0.0022 (10)
C12A	0.0311 (18)	0.0174 (16)	0.0118 (15)	−0.0057 (13)	0.0042 (12)	0.0035 (12)
N4A	0.0159 (13)	0.0142 (13)	0.0087 (12)	0.0000 (10)	0.0008 (9)	−0.0007 (9)
N6B	0.0156 (13)	0.0164 (13)	0.0144 (13)	−0.0020 (10)	0.0031 (9)	−0.0062 (10)
N3B	0.0108 (12)	0.0188 (13)	0.0093 (12)	0.0014 (10)	0.0016 (9)	−0.0030 (10)
O2A	0.0217 (12)	0.0123 (11)	0.0137 (11)	−0.0012 (9)	0.0021 (8)	0.0003 (8)
C12B	0.0272 (17)	0.0241 (17)	0.0065 (14)	0.0013 (14)	0.0013 (12)	−0.0031 (12)
C13B	0.0320 (19)	0.0237 (17)	0.0088 (15)	0.0002 (14)	−0.0002 (12)	0.0033 (12)
C13A	0.0262 (17)	0.0153 (16)	0.0099 (15)	−0.0023 (13)	−0.0010 (12)	−0.0026 (12)
O1A	0.0265 (12)	0.0181 (12)	0.0104 (11)	−0.0025 (9)	0.0016 (9)	0.0019 (8)
N4B	0.0140 (12)	0.0161 (13)	0.0072 (12)	0.0031 (10)	0.0015 (9)	−0.0021 (9)
C5B	0.0097 (14)	0.0194 (16)	0.0076 (14)	0.0019 (11)	0.0000 (10)	−0.0028 (11)
N2A	0.0289 (15)	0.0148 (15)	0.0081 (13)	−0.0034 (11)	0.0021 (10)	0.0000 (10)
N5B	0.0184 (13)	0.0153 (13)	0.0095 (12)	0.0032 (10)	0.0004 (9)	−0.0008 (10)
C11A	0.0109 (14)	0.0122 (14)	0.0129 (14)	0.0015 (11)	0.0043 (10)	−0.0006 (11)
C3A	0.0167 (15)	0.0154 (15)	0.0175 (16)	−0.0018 (12)	0.0053 (12)	−0.0036 (12)
C7A	0.0130 (14)	0.0180 (16)	0.0074 (13)	0.0006 (12)	0.0019 (10)	0.0002 (11)
C5A	0.0091 (14)	0.0148 (15)	0.0124 (14)	0.0004 (11)	0.0043 (10)	−0.0007 (11)
C8B	0.0122 (14)	0.0202 (16)	0.0112 (15)	0.0028 (12)	0.0012 (11)	−0.0030 (12)
C7B	0.0128 (15)	0.0207 (16)	0.0114 (15)	0.0016 (12)	0.0009 (11)	−0.0046 (12)
C1B	0.0114 (15)	0.0189 (17)	0.0185 (16)	0.0023 (12)	0.0014 (11)	−0.0025 (12)
C8A	0.0135 (14)	0.0132 (15)	0.0086 (14)	0.0017 (11)	0.0034 (10)	0.0004 (11)
C2A	0.0155 (15)	0.0185 (16)	0.0123 (15)	−0.0018 (12)	0.0041 (11)	−0.0016 (12)
C6A	0.0106 (14)	0.0139 (15)	0.0139 (15)	0.0018 (11)	0.0041 (11)	0.0013 (11)
C4A	0.0132 (14)	0.0135 (15)	0.0129 (14)	0.0010 (11)	0.0030 (11)	0.0028 (11)
C1A	0.0132 (14)	0.0187 (16)	0.0118 (14)	0.0001 (12)	0.0045 (11)	0.0025 (12)
C3B	0.0090 (13)	0.0178 (16)	0.0080 (14)	0.0031 (11)	0.0016 (10)	−0.0033 (11)
C4B	0.0093 (13)	0.0186 (16)	0.0091 (14)	0.0054 (11)	−0.0001 (10)	−0.0026 (11)
C11B	0.0205 (16)	0.0169 (16)	0.0202 (16)	−0.0051 (13)	0.0054 (12)	−0.0008 (13)
C10B	0.0227 (17)	0.0199 (17)	0.0209 (17)	−0.0036 (13)	0.0043 (13)	−0.0103 (13)
C14A	0.0303 (18)	0.0191 (17)	0.0120 (15)	0.0030 (13)	0.0061 (12)	0.0031 (12)

### Geometric parameters (Å, º)

**Table:**

S1—C5A	1.772 (3)	C13B—N5B	1.475 (4)
S1—C14A	1.802 (3)	C13B—H5A1	0.9800
N1A—C7A	1.282 (4)	C13B—H5A2	0.9800
N1A—C8A	1.391 (4)	C13B—H5A3	0.9800
C6B—N4B	1.329 (4)	C13A—H1'1	0.9800
C6B—N2B	1.377 (4)	C13A—H1'2	0.9800
C6B—C5B	1.409 (4)	C13A—H1'3	0.9800
C10A—O2A	1.225 (3)	N4B—C4B	1.341 (4)
C10A—N4A	1.373 (4)	C5B—C8B	1.455 (4)
C10A—N3A	1.388 (4)	N2A—C11A	1.339 (4)
O1B—C1B	1.203 (4)	N2A—H101	0.85 (4)
C9A—O1A	1.232 (3)	N2A—H102	0.96 (4)
C9A—N4A	1.419 (4)	N5B—C4B	1.369 (4)
C9A—C8A	1.430 (4)	N5B—C1B	1.393 (4)
O2B—C2B	1.220 (3)	C11A—C8A	1.399 (4)
C2B—N6B	1.387 (4)	C3A—C2A	1.391 (4)
C2B—C3B	1.473 (4)	C3A—C4A	1.400 (4)
N3A—C11A	1.379 (4)	C3A—H3	0.9500
N3A—C12A	1.467 (4)	C7A—C6A	1.464 (4)
O3A—C7B	1.213 (3)	C7A—H7	0.9500
N2B—C7B	1.384 (4)	C5A—C4A	1.394 (4)
N2B—C12B	1.472 (4)	C5A—C6A	1.421 (4)
O4A—C8B	1.231 (3)	C2A—C1A	1.388 (4)
N1B—C8B	1.382 (4)	C2A—H4	0.9500
N1B—C7B	1.409 (4)	C6A—C1A	1.397 (4)
N1B—C11B	1.469 (4)	C4A—H2	0.9500
C12A—H2'1	0.9800	C1A—H5	0.9500
C12A—H2'2	0.9800	C3B—C4B	1.420 (4)
C12A—H2'3	0.9800	C11B—H9A1	0.9800
N4A—C13A	1.468 (3)	C11B—H9A2	0.9800
N6B—C1B	1.407 (4)	C11B—H9A3	0.9800
N6B—C10B	1.475 (4)	C10B—H6A1	0.9800
N3B—C3B	1.324 (4)	C10B—H6A2	0.9800
N3B—C5B	1.335 (4)	C10B—H6A3	0.9800
C12B—H8A1	0.9800	C14A—H20A	0.9800
C12B—H8A2	0.9800	C14A—H20B	0.9800
C12B—H8A3	0.9800	C14A—H20C	0.9800
			
C5A—S1—C14A	103.51 (14)	C4B—N5B—C13B	121.4 (2)
C7A—N1A—C8A	124.5 (3)	C1B—N5B—C13B	116.0 (2)
N4B—C6B—N2B	117.7 (2)	N2A—C11A—N3A	117.6 (3)
N4B—C6B—C5B	123.0 (3)	N2A—C11A—C8A	122.2 (3)
N2B—C6B—C5B	119.2 (3)	N3A—C11A—C8A	120.2 (3)
O2A—C10A—N4A	122.0 (3)	C2A—C3A—C4A	120.3 (3)
O2A—C10A—N3A	121.4 (3)	C2A—C3A—H3	119.9
N4A—C10A—N3A	116.6 (2)	C4A—C3A—H3	119.9
O1A—C9A—N4A	118.1 (3)	N1A—C7A—C6A	120.4 (3)
O1A—C9A—C8A	126.1 (3)	N1A—C7A—H7	119.8
N4A—C9A—C8A	115.8 (2)	C6A—C7A—H7	119.8
O2B—C2B—N6B	122.1 (3)	C4A—C5A—C6A	119.5 (3)
O2B—C2B—C3B	123.5 (3)	C4A—C5A—S1	123.0 (2)
N6B—C2B—C3B	114.4 (2)	C6A—C5A—S1	117.6 (2)
C11A—N3A—C10A	122.6 (2)	O4A—C8B—N1B	121.1 (3)
C11A—N3A—C12A	120.4 (2)	O4A—C8B—C5B	123.4 (3)
C10A—N3A—C12A	117.0 (2)	N1B—C8B—C5B	115.5 (2)
C6B—N2B—C7B	122.7 (2)	O3A—C7B—N2B	122.5 (3)
C6B—N2B—C12B	120.2 (2)	O3A—C7B—N1B	120.5 (3)
C7B—N2B—C12B	117.1 (2)	N2B—C7B—N1B	117.0 (2)
C8B—N1B—C7B	124.7 (3)	O1B—C1B—N5B	122.0 (3)
C8B—N1B—C11B	119.1 (2)	O1B—C1B—N6B	121.5 (3)
C7B—N1B—C11B	115.9 (2)	N5B—C1B—N6B	116.5 (3)
N3A—C12A—H2'1	109.5	N1A—C8A—C11A	114.9 (2)
N3A—C12A—H2'2	109.5	N1A—C8A—C9A	125.2 (2)
H2'1—C12A—H2'2	109.5	C11A—C8A—C9A	120.0 (3)
N3A—C12A—H2'3	109.5	C1A—C2A—C3A	119.5 (3)
H2'1—C12A—H2'3	109.5	C1A—C2A—H4	120.3
H2'2—C12A—H2'3	109.5	C3A—C2A—H4	120.3
C10A—N4A—C9A	124.9 (2)	C1A—C6A—C5A	118.8 (3)
C10A—N4A—C13A	115.8 (2)	C1A—C6A—C7A	119.9 (3)
C9A—N4A—C13A	119.3 (2)	C5A—C6A—C7A	121.3 (2)
C2B—N6B—C1B	126.0 (2)	C5A—C4A—C3A	120.4 (3)
C2B—N6B—C10B	118.4 (2)	C5A—C4A—H2	119.8
C1B—N6B—C10B	115.6 (3)	C3A—C4A—H2	119.8
C3B—N3B—C5B	117.2 (2)	C2A—C1A—C6A	121.5 (3)
N2B—C12B—H8A1	109.5	C2A—C1A—H5	119.3
N2B—C12B—H8A2	109.5	C6A—C1A—H5	119.3
H8A1—C12B—H8A2	109.5	N3B—C3B—C4B	121.5 (3)
N2B—C12B—H8A3	109.5	N3B—C3B—C2B	118.4 (2)
H8A1—C12B—H8A3	109.5	C4B—C3B—C2B	120.1 (3)
H8A2—C12B—H8A3	109.5	N4B—C4B—N5B	118.1 (2)
N5B—C13B—H5A1	109.5	N4B—C4B—C3B	121.8 (3)
N5B—C13B—H5A2	109.5	N5B—C4B—C3B	120.1 (3)
H5A1—C13B—H5A2	109.5	N1B—C11B—H9A1	109.5
N5B—C13B—H5A3	109.5	N1B—C11B—H9A2	109.5
H5A1—C13B—H5A3	109.5	H9A1—C11B—H9A2	109.5
H5A2—C13B—H5A3	109.5	N1B—C11B—H9A3	109.5
N4A—C13A—H1'1	109.5	H9A1—C11B—H9A3	109.5
N4A—C13A—H1'2	109.5	H9A2—C11B—H9A3	109.5
H1'1—C13A—H1'2	109.5	N6B—C10B—H6A1	109.5
N4A—C13A—H1'3	109.5	N6B—C10B—H6A2	109.5
H1'1—C13A—H1'3	109.5	H6A1—C10B—H6A2	109.5
H1'2—C13A—H1'3	109.5	N6B—C10B—H6A3	109.5
C6B—N4B—C4B	115.6 (2)	H6A1—C10B—H6A3	109.5
N3B—C5B—C6B	120.9 (3)	H6A2—C10B—H6A3	109.5
N3B—C5B—C8B	118.4 (2)	S1—C14A—H20A	109.5
C6B—C5B—C8B	120.8 (3)	S1—C14A—H20B	109.5
C11A—N2A—H101	116 (2)	H20A—C14A—H20B	109.5
C11A—N2A—H102	125 (2)	S1—C14A—H20C	109.5
H101—N2A—H102	118 (3)	H20A—C14A—H20C	109.5
C4B—N5B—C1B	122.6 (2)	H20B—C14A—H20C	109.5
			
O2A—C10A—N3A—C11A	179.6 (3)	C8B—N1B—C7B—N2B	2.3 (4)
N4A—C10A—N3A—C11A	−1.0 (4)	C11B—N1B—C7B—N2B	175.0 (2)
O2A—C10A—N3A—C12A	0.8 (4)	C4B—N5B—C1B—O1B	178.7 (3)
N4A—C10A—N3A—C12A	−179.7 (3)	C13B—N5B—C1B—O1B	−1.7 (4)
N4B—C6B—N2B—C7B	178.6 (3)	C4B—N5B—C1B—N6B	−1.3 (4)
C5B—C6B—N2B—C7B	−2.0 (4)	C13B—N5B—C1B—N6B	178.3 (3)
N4B—C6B—N2B—C12B	−0.9 (4)	C2B—N6B—C1B—O1B	−174.9 (3)
C5B—C6B—N2B—C12B	178.5 (3)	C10B—N6B—C1B—O1B	3.4 (4)
O2A—C10A—N4A—C9A	178.1 (3)	C2B—N6B—C1B—N5B	5.1 (4)
N3A—C10A—N4A—C9A	−1.3 (4)	C10B—N6B—C1B—N5B	−176.6 (2)
O2A—C10A—N4A—C13A	−2.4 (4)	C7A—N1A—C8A—C11A	−179.1 (3)
N3A—C10A—N4A—C13A	178.1 (2)	C7A—N1A—C8A—C9A	0.5 (5)
O1A—C9A—N4A—C10A	−178.9 (3)	N2A—C11A—C8A—N1A	−2.3 (4)
C8A—C9A—N4A—C10A	1.7 (4)	N3A—C11A—C8A—N1A	177.4 (2)
O1A—C9A—N4A—C13A	1.6 (4)	N2A—C11A—C8A—C9A	178.1 (3)
C8A—C9A—N4A—C13A	−177.7 (2)	N3A—C11A—C8A—C9A	−2.2 (4)
O2B—C2B—N6B—C1B	174.2 (3)	O1A—C9A—C8A—N1A	1.2 (5)
C3B—C2B—N6B—C1B	−6.4 (4)	N4A—C9A—C8A—N1A	−179.5 (3)
O2B—C2B—N6B—C10B	−4.1 (4)	O1A—C9A—C8A—C11A	−179.2 (3)
C3B—C2B—N6B—C10B	175.4 (2)	N4A—C9A—C8A—C11A	0.1 (4)
N2B—C6B—N4B—C4B	179.0 (2)	C4A—C3A—C2A—C1A	−0.8 (4)
C5B—C6B—N4B—C4B	−0.4 (4)	C4A—C5A—C6A—C1A	−0.8 (4)
C3B—N3B—C5B—C6B	−0.6 (4)	S1—C5A—C6A—C1A	178.6 (2)
C3B—N3B—C5B—C8B	178.9 (3)	C4A—C5A—C6A—C7A	179.9 (3)
N4B—C6B—C5B—N3B	0.5 (4)	S1—C5A—C6A—C7A	−0.8 (4)
N2B—C6B—C5B—N3B	−178.9 (3)	N1A—C7A—C6A—C1A	−1.0 (4)
N4B—C6B—C5B—C8B	−179.0 (3)	N1A—C7A—C6A—C5A	178.3 (3)
N2B—C6B—C5B—C8B	1.6 (4)	C6A—C5A—C4A—C3A	1.2 (4)
C10A—N3A—C11A—N2A	−177.6 (3)	S1—C5A—C4A—C3A	−178.1 (2)
C12A—N3A—C11A—N2A	1.1 (4)	C2A—C3A—C4A—C5A	−0.5 (4)
C10A—N3A—C11A—C8A	2.7 (4)	C3A—C2A—C1A—C6A	1.2 (4)
C12A—N3A—C11A—C8A	−178.6 (3)	C5A—C6A—C1A—C2A	−0.5 (4)
C8A—N1A—C7A—C6A	179.3 (3)	C7A—C6A—C1A—C2A	178.9 (3)
C14A—S1—C5A—C4A	−0.3 (3)	C5B—N3B—C3B—C4B	0.6 (4)
C14A—S1—C5A—C6A	−179.7 (2)	C5B—N3B—C3B—C2B	−178.9 (2)
C7B—N1B—C8B—O4A	177.1 (3)	O2B—C2B—C3B—N3B	3.0 (4)
C11B—N1B—C8B—O4A	4.6 (4)	N6B—C2B—C3B—N3B	−176.5 (2)
C7B—N1B—C8B—C5B	−2.6 (4)	O2B—C2B—C3B—C4B	−176.5 (3)
C11B—N1B—C8B—C5B	−175.1 (3)	N6B—C2B—C3B—C4B	4.1 (4)
N3B—C5B—C8B—O4A	1.4 (4)	C6B—N4B—C4B—N5B	−179.7 (2)
C6B—C5B—C8B—O4A	−179.1 (3)	C6B—N4B—C4B—C3B	0.4 (4)
N3B—C5B—C8B—N1B	−178.9 (2)	C1B—N5B—C4B—N4B	179.6 (3)
C6B—C5B—C8B—N1B	0.6 (4)	C13B—N5B—C4B—N4B	0.0 (4)
C6B—N2B—C7B—O3A	−179.0 (3)	C1B—N5B—C4B—C3B	−0.5 (4)
C12B—N2B—C7B—O3A	0.4 (4)	C13B—N5B—C4B—C3B	179.9 (3)
C6B—N2B—C7B—N1B	0.2 (4)	N3B—C3B—C4B—N4B	−0.5 (4)
C12B—N2B—C7B—N1B	179.6 (2)	C2B—C3B—C4B—N4B	178.9 (2)
C8B—N1B—C7B—O3A	−178.5 (3)	N3B—C3B—C4B—N5B	179.6 (3)
C11B—N1B—C7B—O3A	−5.8 (4)	C2B—C3B—C4B—N5B	−0.9 (4)

### Hydrogen-bond geometry (Å, º)

**Table:**

*D*—H···*A*	*D*—H	H···*A*	*D*···*A*	*D*—H···*A*
N2*A*—H101···N1*A*	0.85 (4)	2.32 (3)	2.701 (4)	107 (2)
N2*A*—H101···O2*B*^i^	0.85 (4)	2.37 (3)	3.078 (3)	141 (3)
N2*A*—H102···O4*A*^i^	0.96 (4)	2.29 (4)	3.238 (3)	169 (3)
C12*A*—H2′1···O2*A*	0.98	2.25	2.706 (4)	107
C4*A*—H2···O2*A*^ii^	0.95	2.58	3.159 (4)	119
C3*A*—H3···O2*A*^ii^	0.95	2.49	3.114 (4)	123
C12*A*—H2′3···O4*A*^i^	0.98	2.36	3.014 (4)	123
C1*A*—H5···O2*B*^i^	0.95	2.45	3.339 (4)	155
C12*B*—H8*A*2···O3*A*	0.98	2.33	2.720 (4)	103
C7*A*—H7···S1	0.95	2.59	3.017 (3)	108
C7*A*—H7···O1*A*	0.95	2.18	2.849 (3)	127
C13*B*—H5*A*1···O2*B*^i^	0.98	2.50	3.315 (4)	140
C13*B*—H5*A*3···N4*B*	0.98	2.34	2.770 (4)	106
C13*A*—H1′2···O1*A*	0.98	2.28	2.726 (4)	107
C11*B*—H9*A*1···O4*A*	0.98	2.37	2.744 (4)	102
C10*B*—H6*A*3···O2*B*	0.98	2.31	2.751 (4)	107
C10*B*—H6*A*1···O1*A*^iii^	0.98	2.58	3.542 (4)	169

Symmetry codes: (i) *x*+1/2, −*y*+1/2, *z*+1/2; (ii) −*x*+3/2, *y*+1/2, −*z*+1/2; (iii) *x*−1, *y*, *z*.
